# BDNF and Krox20 as Indicators of Platelet-rich Plasma-Induced Nerve Regeneration in a Neuropathic Orofacial Pain Model

**DOI:** 10.1055/s-0043-1761194

**Published:** 2023-06-13

**Authors:** Saka Winias, Andari Sarasati, Satutya Wicaksono, Nurina Febriyanti Ayuningtyas, Diah Savitri Ernawati, Desiana Radithia

**Affiliations:** 1 Department of Oral Medicine, Faculty of Dental Medicine, Universitas Airlangga, Surabaya, Indonesia; 2 Faculty of Dental Medicine, Universitas Airlangga, Surabaya, Indonesia

**Keywords:** peripheral nerve injury, neuro regeneration, BDNF, PRP, Krox20

## Abstract

**Objective** Various growth factors contained in PRP can increase angiogenesis and cell proliferation, which plays an essential role in the process of neuroregeneration and peripheral nerve injury recovery. This study analyzed PRP effects in the neuro-regeneration of axonotmesis through brain-derived neurotrophic factor (BDNF) and Krox20 expressions.

**Materials and Methods** Freeze-dried allogeneic platelet-rich plasma (PRP) were prepared from allogeneic sources. Forty-two *Rattus norvegicus* were divided into three groups: negative control group, positive control group (crushing infraorbital nerve) and treatment group (crushing infraorbital nerve without PRP injection). Each group was observed for fourteen and twenty-one days after injury. Infraorbital nerve tissue is isolated for indirect immunohistochemistry examination with BDNF and Krox20 antibodies. Data analysis was performed using One-Way ANOVA and Mann-Whitney tests with significant value as p < 0.05.

**Results** The PRP group showed BDNF expression significantly higher than control positive groups, both observation days (p = 0.00). A higher Korx20 expression showed by the PRP group after 21 days than in the control positive groups (p = 0.002).

**Conclusion** PRP can potentially improve neuroregeneration of axonotmesis through increased BDNF and Krox20 expression on the twenty-one days after injury.

## Introduction

Orofacial pain (OFP) is a general terminology for pain that occurs in the orofacial region, which includes the oral cavity, jaw, and face. OFP was reported to occur in 27.5% of the general population, 1 and Indonesia's population 49.9%. 2 3 Symptoms of OFP can be caused by abnormalities in the musculoskeletal, nervous, psychological or intracranial system. 4 OFP that occurs due to lesions or diseases of the somatosensory system is called neuropathic pain (NP). NP is suffered by 7-8% of the general population, of which 20-25% classify the pain as chronic. 5 A study conducted in Indonesia revealed that cases of neuropathic pain were higher than nociceptive pain (51.3%, and 41.9%; respectively). 6

Drug consumption in NP management is known to be palliative, which is not efficient enough and causes various side effects. 7 NP is also caused by the inability of the nerve to regenerate its anatomy adequately; thus, regenerative therapy is needed to specifically improve neuroregeneration to restore anatomical structure and relieve pain. 8 Nerve repair has been clinically achieved through microsurgery and autograph nerve transplants. However, this therapy cannot recreate the cellular and molecular environment of the nerve, so nerve function does not fully recover. 9

Successful axonal regeneration and reinnervation of nerve fibers depend on cellular and molecular responses to the nerve injury. These responses involve debris clearance by macrophages and Schwann cells (SCs) phenotype plasticity into a repair cell type that leads to the activation of various proteins and neurotrophic factors such as brain-derived neurotrophic factor (BDNF) to induce axonal elongation. However, neurotrophic factors secretion and SC proliferation are known to decrease in a tapering manner along with the axonal regeneration rate caused by the signalling changes. This condition leads to imperfect axonal elongation and unsuccessful nerve functional recovery in chronic nerve injury cases. An altered signalling pathway of SC plasticity and axonal regeneration could lead to improper remyelination activation and myelin sheath maintenance regulated by the Krox20 protein. Constant and adequate stimulation of SC proliferation is needed to promote inflammatory responses and neurotrophic factors secretion. 10

Platelet-rich plasma (PRP) is commonly used in clinical musculoskeletal therapy. PRP was developed to regenerate tissue by utilizing growth factors in α-granule platelets. Various growth factors contained in PRP can increase angiogenesis and cell proliferation that affect the viability and activity of SC in producing neurotrophic elements to produce myelin, which plays an essential role in the process of neuro-regeneration and nerve functional recovery. 9 Previous in-vivo studies observed the PRP neuroregeneration effect on sciatic nerve injury in rats, but its impact on rat infraorbital nerve injuries has not yet been observed. Observation of SC activity through BDNF and Krox20 expression is expected to describe the potential of PRP in increasing the success of infraorbital nerve regeneration to relieve orofacial neuropathic pain.

## Materials and Methods

### Animal

This research is an experimental laboratory with a post-test-only control group design. Forty-two healthy male Wistar rats ( *Rattus norvegicus* ) weighing 200-300 grams were selected. The protocol of this study has been registered and approved by the Ethical Clearance Commission of the Faculty of Dental Medicine, Universitas Airlangga, with registration number 270/HRECC.FODM/V/2019.

### Preparation of Platelet-rich Plasma (PRP)

**Fig. 1:**
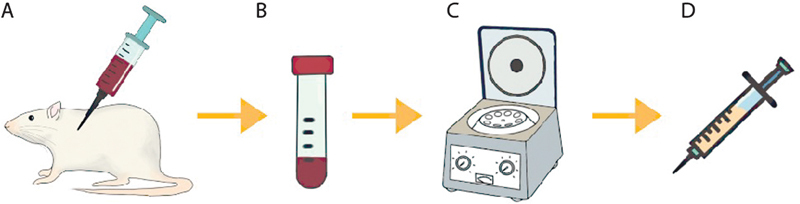
PRP preparation. ( **A** ) Blood extraction; ( **B** ) Extracted blood; ( **C** ) Centrifugation; ( **D** ) Injectable PRP + CMC 1% solution.

Ten Wistar rats ( *Rattus norvegicus* ) were used to extract their blood from its heart to make PRP, as shown in Figure 1 . The extracted blood was centrifuged *(Corelab BLC – 2012)* for ten minutes at 4000rpm twice. The centrifuged blood was then preserved, frozen at -83°C for 12 hours, and lyophilized *(Virtis Benchtop 4K 4BT4K2L-105)* for 8-12 hours. This process resulted in the formation of freeze-dried platelet-rich plasma, which was then sterilized by UV light. Then the PRP was mixed with carboxymethylcellulose (CMC) 1% (ratio 1:1).

### Neuropathic Orofacial Pain Model

**Fig. 2:**
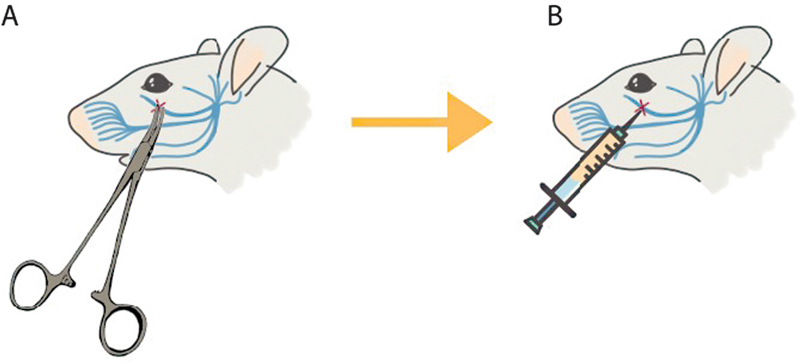
Sample treatment. ( **A** ) Infraorbital nerve crushing using artery clamp; ( **B** ) PRP injection on crushing site 24 hours post-crushing.

The neuropathic orofacial pain model was created with a surgical approach. The infraorbital nerve of Rattus norvegicus was crashed using an artery clamp in the most vital key position for 15 seconds ( Figure 2A ). During the procedure, ketamine (100 mg/kg) was used as anesthesia and administered intraperitoneally.

### Research Model Treatment

The animal was divided into three groups:

1. Negative control group, normal condition
2. Positive control, axonotmesis injury model
3. Treatment, axonotmesis injury model and treated by the PRP (0.1 ml) the 24 hours post-induction ( Figure 2B ).

Each group was then observed and euthanised using a lethal dose of ketamine intraperitoneally on the fourteen and twenty-first day of treatment. The infraorbital nerve tissue was then taken for stain processing.

### BDNF and Krox-20 Expression

Immunohistochemical staining was done on the infraorbital nerve tissue using monoclonal anti-BDNF *(Bioss, bsm-52368R, United States)* and polyclonal anti-Krox20 primary antibodies *(Antibody Online, ABIN2927054, Germany)* . The specified expressions were then observed based on the number of expressed SCs in the field of view of the ImageJ application and a light microscope *(Nikon Eclipse e200)* under 400x magnification.

### Statistical Analysis

Statistical Package for the Social Sciences 20.0 software (SPSS for Windows, SPSS, Chicago, USA) was used to express and analyze data. Data were expressed as mean ± standard deviation (SD). Data were analyzed with Kolmogorov-Smirnov followed by the Levene test. Statistical analysis for BDNF expression data was performed by a one-way analysis variance test (ANOVA) with Tukey's HSD ( *p* < 0.05). Meanwhile, statistical analysis for Krox20 expression data was performed using the Mann-Whitney test ( *p* < 0.05).

## Results

### BDNF Expression

**Fig. 3:**
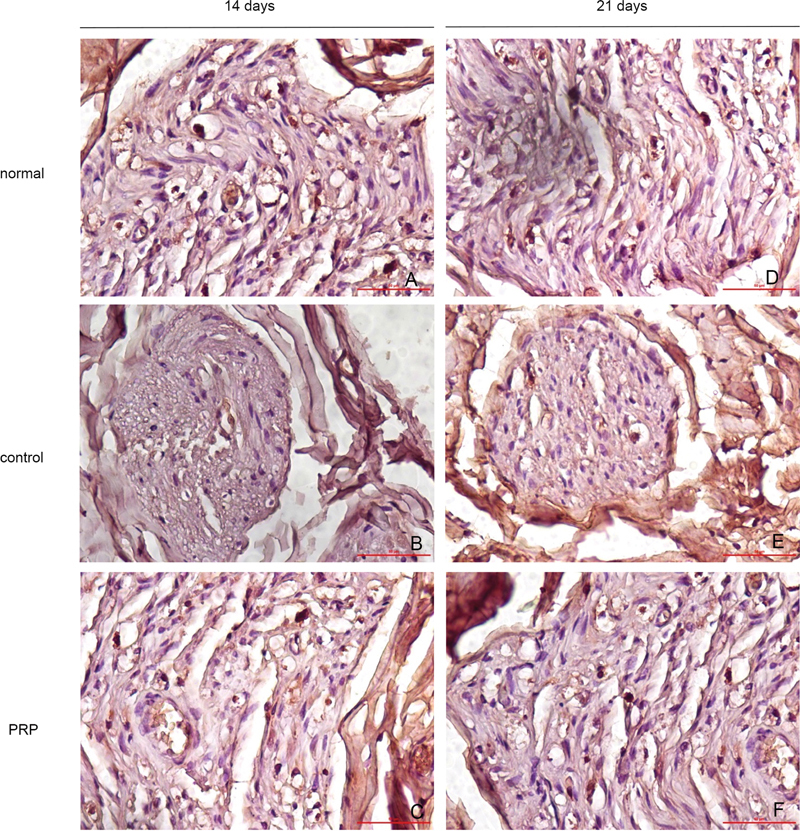
The BDNF was expressed on SC. The micrographs were taken under 400x magnifications of the light microscope.

**Fig. 4:**
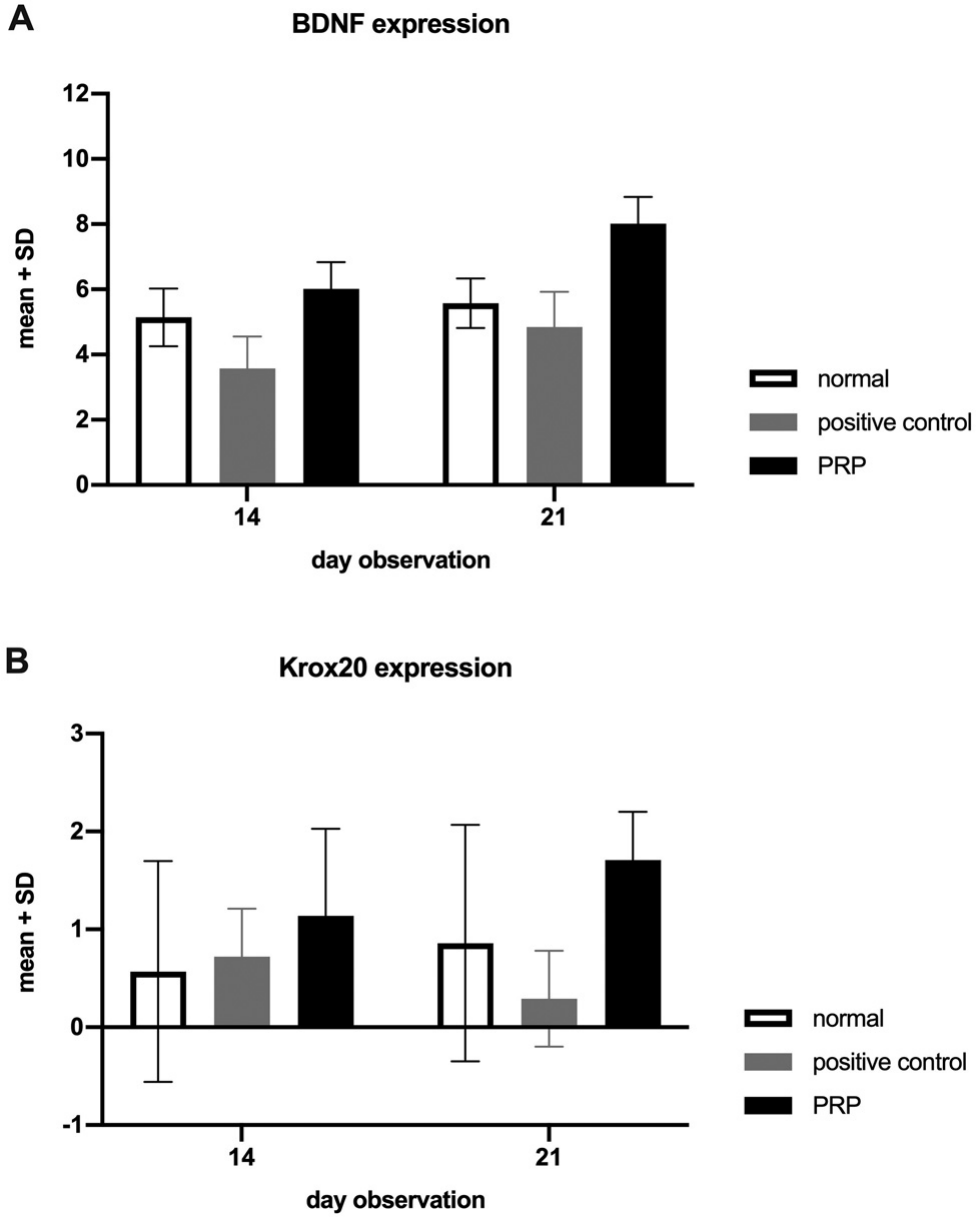
The mean value of BDNF expression ( **A** ) and Korx20 expression ( **B** ).

The BDNF expression in the SC showed in Figure 3 . The PRP group showed BDNF expression in 14 days significantly higher than control positive groups (p = 0.00). A higher BDNF expression was also showed by the PRP group after 21 days than in control positive groups and normal groups (p = 0.000) ( Figure 4A ).

### Krox20 Expression

**Fig. 5:**
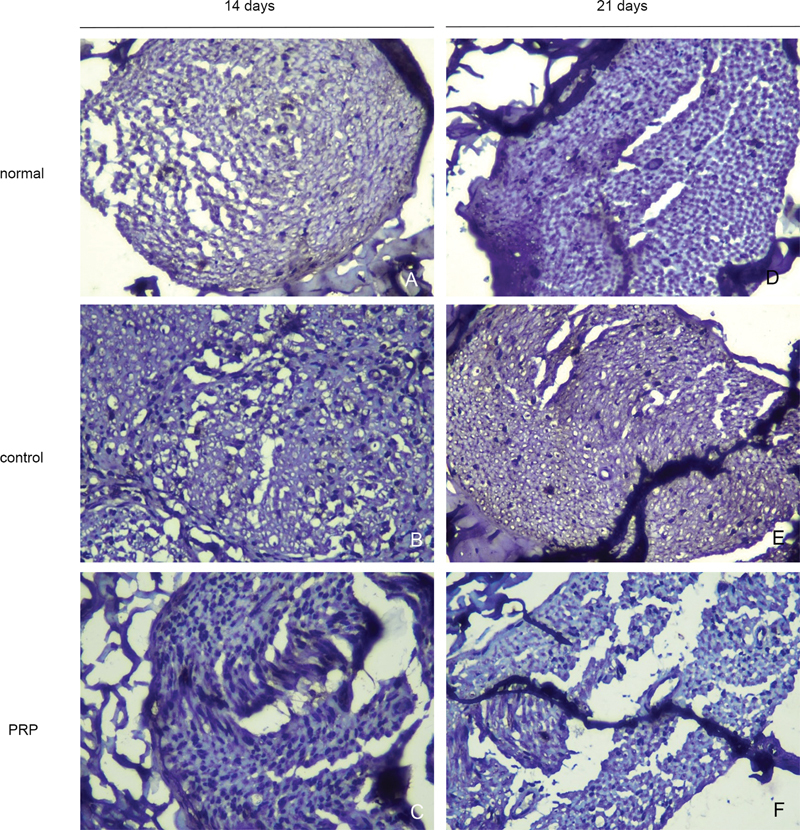
The Korx20 was expressed on SC. The micrographs were taken under 400 x of the light microscope.

The Krox20 expression in the SC showed in Figure 5 . The PRP group showed Korx20 expression in 14 days not different from the normal and positive control (p = 0.19 and p = 0.298, respectively). A higher Korx20 expression showed by the PRP group after 21 days than in the control positive groups (p = 0.002) ( Figure 4B ).

## Discussion

This study analyzed PRP effects on neuro-regeneration to relieve crush-induced orofacial neuropathic pain. The authors found that PRP administration escalates expressions of BDNF and Krox20 the 21 days after crush injury, implying an increased neuroregeneration rate and neuroinflammation resolution caused by the immune response to axonotmesis injury and various signalling activity. These results align with previous studies suggesting the benefits of PRP administration to neuroregeneration.

Axonotmesis on an infraorbital nerve induces an innate neuroinflammation response around the injured nerve tissue, defined as Wallerian degeneration (WD). 11 12 13 In peripheral nerve regeneration, BDNF can promote the viability and proliferation of neurons and SCs and induce axonal elongation via an autocrine and/or paracrine manner. BDNF acquires its pro-regenerative quality through the binding activity towards tropomyosin receptor kinase-B (TrkB). 14 BDNF/TrkB signalling activates three central downstream phosphorylation cascades, PLC; PI3K; MAPK/ERK pathway. 15 16 17 Another study by Grosheva *et al.* reported that a better axon structural and functional outcome of an injured-facial nerve is followed by increased BDNF secretion from neurons and SCs. 18

The present study revealed substantial results that indicate the administration of PRP may enhance the neuro-regeneration of an injured-infraorbital nerve. This deductive approach stated a substantial difference of BDNF expression between 14 and 21 days ( *p* = 0.04; p < 0.05). Meanwhile an insignificant escalation of BDNF expression was found in the control positive group both 14 and 21 days ( *p* = 0.127; p > 0.05). The condition is also supported by the results, revealing a significant difference in BDNF expression between positive and PRP groups in both 14 and 21 days ( *p* = 0.000; p < 0.05). Five main growth factors in PRP may induce BDNF expression by stimulating the proliferative and neurotrophic activity of SCs. These growth factors are intrinsic neurotrophic factors in response to injury in the peripheral nerve. Fibroblast growth factors (FGF), through their binding with FGF receptors (FGFR), can generate downstream signal transduction resulting in various neurotrophic activities. 19 20 Insulin-like growth factors (IGFs) I and II are potent neuronal mitogens and survival factors. IGF-I/IGF-1R signaling pathways induce the phosphorylation of MAPK/phosphatidylinositol-3 kinase (PI3K). This signaling pathway activation may enhance the viabilities of SCs and neurons. 21 22 23 24 Other substances, such as platelet-derived growth factor (PDGF), vascular endothelial growth factor (VEGF), and transforming growth factor-β (TGF-β), also exert anti-apoptotic and neuroprotective activity toward SCs and neurons. 20 Neuroprotective and neuro-regenerative exerted by GFs in PRP may indirectly result in a substantial escalation of BDNF expression through the enhancement of SCs' innate neurotrophic properties.

Remyelination occurs on fully regenerated axons after WD is complete, along with decreased c-Jun expression. 25 Extracellular signals like growth factors, cytokines, injury and cellular stress induce intracellular pathways. Unbalanced action potentials caused by nerve injury and inflammation could induce cAMP, whereas the remyelination process is cAMP-dependent. cAMP enhancement in SC will activate Krox20 signalling and expression through the effector exchange protein activated by cyclic AMP (EPAC) activation. This leads to SC differentiation to myelinating SC and inhibits the c-Jun NH2-terminal protein kinase (JNK)-the c-Jun pathway to prevent further immune response. 26 27 Krox20 is the main transcription factor in myelin formation by producing myelin proteins such as myelin basic protein (MBP), peripheral myelin protein 22 (PMP22) and myelin protein zero (MPZ). 28 This proves that Krox20 sufficiently acts as a remyelination marker. Krox20 expression in the control positive group and PRP groups shows insignificant results ( *p* = 0.298). Previous studies state that WD is resolved after the 14 ^th^ day after the injury or after debris has been fully phagocyted. 12 Thus, the SC phenotype transition from non-myelinating to myelinating is suspected of not fully occurring by the 14 ^th^ day. Therefore, the expression of c-Jun was thought to be more dominant than the Krox20 expression. The previous study also implicated a stagnant increase in Krox20 expression on the 14 ^th^ day after injury. 29 This suggests that PRP's neuroinflammation acceleration has not reached an ideal resolution by the 14 ^th^ day after injury.

This study shows significant results of Krox20 expression between control positive groups and PRP groups ( *p* = 0.002). Although insignificant, decreased Krox20 expression after PRP treatment might play a role in the result. This study shows that Krox20 expression is already activated on the 14 ^th^ day after the injury. However, an absence of anti-inflammatory acceleration from exogenous growth factors in the PRP might cause imperfect neuroinflammation and decrease Krox20 signalling. Excessive inflammatory conditions due to a crush injury can cause proteases to no longer work selectively against debris but also degrade growth factors, and receptors and prevent other inflammatory cells from functioning normally. Previous studies state that pro-inflammatory cytokines expression still existed on the 21 ^st^ day; decreasing Krox20 and MPZ expression confirmed this result. 30 31

Significant escalation of Krox20 expression between the positive control group and PRP group 21 days after treatment indicates that the PRP can mediate neuroinflammation and accelerate neuro-regeneration but has no direct stimulation to myelin formation. These effects come from GFs like IGF-1, PDGF, TGF-β and VEGF that act as anti-inflammatory agents, survival factors and mitotic agents, as mentioned above. These growth factors directly increase angiogenesis and accelerate SC proliferation and migration, which results in increased production and activation of neurotrophic factors such as BDNF-Trk signaling that will lead to more rapid remyelination by enhancing Krox20 activation through Erk1/2 signalling to secrete myelin proteins. 9 18 32 33 34

This study is limited by a short observation period until 21 days after the injury, so it can't describe the inflammation resolution and peak of neuro-regeneration on the following days. Future research is required to observe the number of inflammatory cells and the expression of BDNF and Krox20 furthers and is also needed through continued behavioral, cellular and molecular observation. The application of PRP is also important to discuss in the future, both injection and applied during the surgery.

## Conclusion

This study confirms that PRP improves the neuroregeneration of infraorbital nerve axonotmesis injury through increased BDNF and Krox20 expression. This is demonstrated by the significant difference observed on the 21 day after axonotmesis injury and PRP treatment.
